# Changing Attitudes towards Occupational Medicine with Blended Learning Methods Is Possible among Medical Students in Spain: A Longitudinal Study

**DOI:** 10.3390/ijerph19020878

**Published:** 2022-01-13

**Authors:** Isabel Iguacel, Begoña Abecia, José Luis Bernal, Begoña Martínez-Jarreta

**Affiliations:** 1 Faculty of Health Sciences, University of Zaragoza, 50009 Zaragoza, Spain; 2 Instituto Agroalimentario de Aragón, 50013 Zaragoza, Spain; 3 Instituto de Investigación Sanitaria Aragón, 50009 Zaragoza, Spain; mjarreta@unizar.es; 4 Centro de Investigación Biomédica en Red de Fisiopatología de la Obesidad y Nutrición, 50009 Zaragoza, Spain; 5 Faculty of Education, University of Zaragoza, 50009 Zaragoza, Spain; begoabecia@gmail.com (B.A.); jbernal@unizar.es (J.L.B.); 6 Faculty of Medicine, University of Zaragoza, 50009 Zaragoza, Spain

**Keywords:** occupational health, blended learning, attitudes, medical students, e-learning

## Abstract

Medical students generally express a low interest in Occupational Medicine. We aimed to assess the attitudes and changes in attitudes of students towards this area after completing a course on Occupational Medicine in two Medical Universities in Spain (Zaragoza and Castilla-La Mancha). The teaching method included blended learning as a model that used online virtual patient platforms (CASUS) and/or EMUTOM, as well as traditional methods such as face-to-face teaching. A total of 526 students (98 of whom attended the University of Castilla-La Mancha) participated during three academic years (2015–2016, 2016–2017 and 2017–2018). The validation of the questionnaire was carried out using reliability, exploratory and confirmatory factor analysis. For the analysis of internal consistency and discrimination, Cronbach’s alpha was used. The adequacy of the factor analysis was measured by means of KMO, and a correlation matrix was examined by means of Bartlett’s test of sphericity. To identify differences between students before and after completing the course, the Mann–Whitney U-test for independent samples was used. Our results show that despite a negative or neutral attitude towards Occupational Medicine, the acquisition of competences and skills in this area and their training were recognized as fundamental for their future professional performance as doctors in any specialty.

## 1. Introduction

New technologies provide training opportunities for students with different and distant geolocations or who are unable to attend classes due to time/place constraints or medical conditions [1]. Despite the substantial development of educational technology tools triggered by the COVID-19 pandemic situation, the use of traditional resources, such as a master class taught by a lecturer, still predominates.

There are three training models that can be defined depending on the learning method: face-to-face, blended and distance learning [1]. Among these models, one method has attracted particular attention in recent years for combining traditional and innovative teaching methods and using both physical and virtual classrooms. This method is known as blended learning [1], blended-e-learning [2] or hybrid learning [3].

Students have a very positive attitude towards the prospect of being taught in a blended learning format and the possibility of making use of innovative teaching tools [4,5]. Nevertheless, the evidence of student satisfaction with blended learning educational models is limited and based on very different contexts and samples, which means that their results are not directly comparable.

The verification of a change in a student’s attitude after learning a subject is an aspect of great interest in areas focused on perception bias or a prejudice that needs to be eliminated. Occupational Medicine, whose purpose is the prevention of illnesses and accidents resulting from work, has long been relegated to the background relative to other medical specialties (cardiology, neurosurgery, gynecology, etc.) that have acquired importance in the public eye, despite its origins in antiquity. Some of the reasons for this negative attitude given by medical students are the fact that Occupational Medicine is detached from clinical practice and diagnosis and is more related to administrative tasks [6].

The COVID-19 pandemic has highlighted the strategic role of occupational physicians at hospitals and workplaces. However, Occupational Medicine faces many future challenges due to a lack of OM specialists and the limited Occupational Medicine teaching and training received by medical students at some European Medical Faculties [6]. Despite all of the efforts made to promote this specialty, the key role that Occupational Medicine plays is still not appropriately appreciated. Education and training are highly valuable tools to change the perceptions and attitudes of students. The specialty of Occupational Medicine (OM) is still undervalued by doctors and is not clearly recognized as an exceptionally important and influential medical discipline that produces transversal knowledge and skills that are necessary for general medicine and most medical specialties.

A blended learning model has been used as a learning tool to teach the subject of Occupational Medicine in medical degree courses in some universities in Spain. The teaching method for this subject includes traditional teaching tools, such as lectures, together with other innovative methods freely accessible on the Web, whose materials (contents, videos, documents, applications, available tests, etc.) can be used autonomously by the student. These innovative methods include the two platforms known as CASUS or virtual patient platform (a problem-based learning (PBL) application with approximately 200 virtual occupational medicine patient cases for teaching use) and the e-learning module EMUTOM (European Module for Undergraduate Education in Occupational Medicine), which compiles materials, cases and resources and makes them accessible online, and its contents have been considered the minimum for learning the subject of Occupational Medicine by future doctors within the framework of the European Union [7,8].

The study of possible changes in the attitudes of students who decided to take the Occupational Medicine course could be of interest from a medical and educational perspective. Consequently, the present study aimed to assess the attitudes and changes in attitudes of a sample of university students taught with two different teaching innovative tools and with a blended learning teaching-learning model for teaching the subject of Occupational Medicine. This ideal scenario allows the verification of the effect of using these innovative methods on the modification of perceptions and prejudices towards a subject.

## 2. Materials and Methods

**Figure 1:**
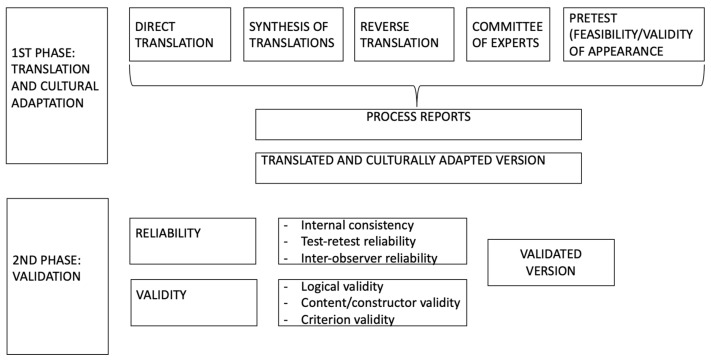
Process of translation and validation of the adapted questionnaire used by Smits and Verbeek [9].

**Figure 2:**
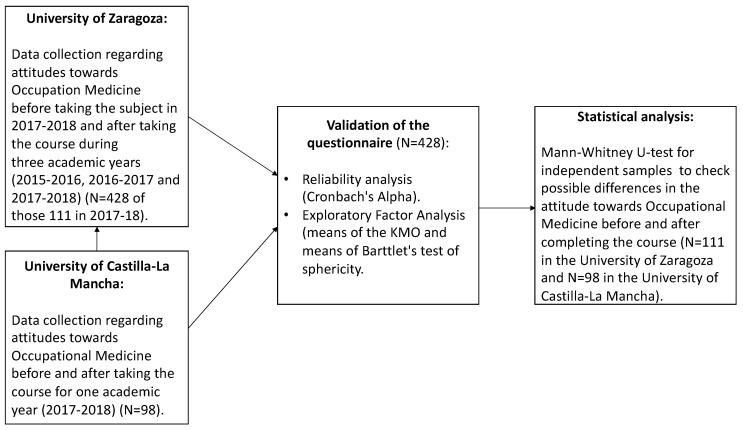
Sample size.

**Figure 3:**
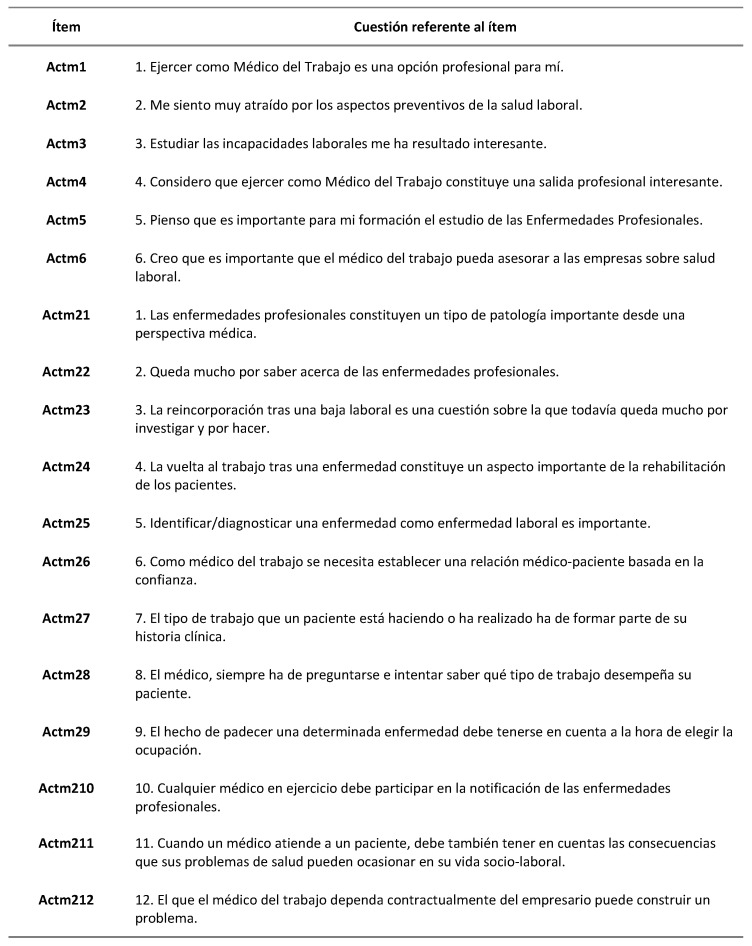
Questionnaire used by Smits and Verbeek adapted to Spanish students. Each of the items (18 items) corresponds to 1 of the 18 questions, all of which take ordinal values [9] A career in Occupational Medicine (six statements), 1. The job of the occupational physician is challenging (Actm1), 2. I am very much attracted to the preventive aspects of occupational health (Actm2), 3. Occupational disability is an interesting topic to study (Actm3), 4. One of my options is to become an occupational physician (Actm4), 5. I find it interesting to study occupational diseases (Actm5), 6. Advising companies about healthy work is attractive to me (Actm6); Occupational Medicine as an interesting medical specialty (11 statements), 1. From the clinical point of view, occupational diseases are not very interesting (reversed coding) (Actm21), 2. There is still a lot to be discovered about occupational diseases (Actm22), 3. There is still a lot to be discovered about return to work after illness (Actm23), 4. Return to work after illness forms an important part of rehabilitation (Actm24), 5. It is important to diagnose an illness as an occupational disease (Actm25), 6. As an occupational physician you need trust between you and your patient (Actm26), 7. The kind of work a patient is doing forms an important part of the medical history (Actm27), 8. As a physician, you will always know what kind of job your patient holds (Actm28),9. If you have a certain illness, it is important to take this into consideration already when choosing a career (Actm29), 10. Every physician should report occupational diseases (Actm210), 11. With every patient, the doctor looks at the complaints but also at its consequences for functioning in daily life and at work (Actm211); Role and position of the occupational physician (one statement), 12. It is a problem that, as an occupational physician, one is not independent from the employer (Actm212).

The questionnaire of Smits and Verbeek was used to measure medical students’ attitudes towards occupational medicine [9]. The questionnaire, consisting of 18 items, was previously applied in a sample of Dutch medical students. Therefore, it was translated into Spanish, back translated, and adapted to the context of Spanish students by a group of experts in Occupational Medicine (Figure 1, Figure 2 and Figure 3).

The validation of the questionnaire, which takes ordinal values on a scale of 1 to 5, was carried out using reliability analysis and exploratory and confirmatory factor analysis [10]. For the analysis of the internal consistency and discrimination capacity of the questionnaire items, Cronbach’s alpha was used.

The adequacy of the factor analysis was measured by means of KMO, and the correlation matrix was examined by means of Bartlett’s test of sphericity.

In addition, to determine whether there were significant differences in attitudes towards Occupational Medicine before and after completing the course, a bivariate study was carried out. As the variables (items) had quantitative values, the nonparametric technique of the Mann–Whitney U-test for independent samples was used. The Student’s *t*-test for independent samples was not applied since the hypotheses did not meet the requirements for the normality of data and equality of variances. The confidence level chosen for the different tests was 95%.

The statistical package SPSS 20.0 was used to carry out the mathematical/statistical analysis.

Finally, the questionnaire included some open questions regarding the perception of blended learning, satisfaction with the subject of Occupational Medicine in relation to its blended learning format and with innovative teaching tools used in the course, the perception of the CASUS tool and the EMUTOM module, changes in attitudes towards the subject of Occupational Medicine after taking the course and the disadvantages of the traditional methodology compared to a blended learning format. In particular, the following open questions were included: “What do you think about the blended learning method? That is, the education that combines online educational materials and opportunities for interaction online with traditional place-based classroom methods”. “Are you satisfied with the subject of Occupational Medicine in relation to its blended learning format?” “Are you satisfied with innovative teaching tools used in the degree?” “What is your perception about the Casus tool?” “What is your perception about the EMUTOM tool?” “Has your attitude changed after completing the subject of Occupational Medicine?” “What do you think are the disadvantages of the traditional methodology compared to a blended learning format?”.

### 2.1. Attitudes towards Occupational Medicine after Completing the Course

**Figure 4:**
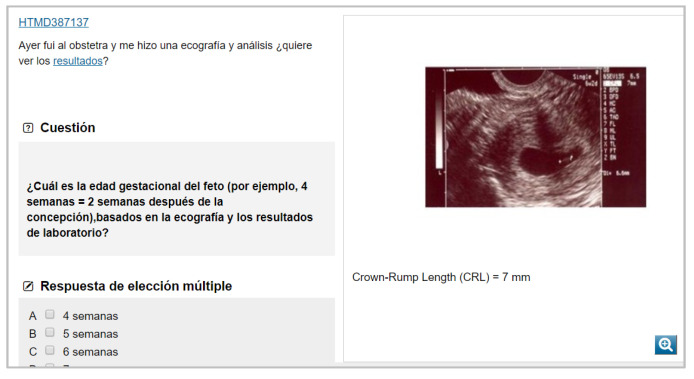

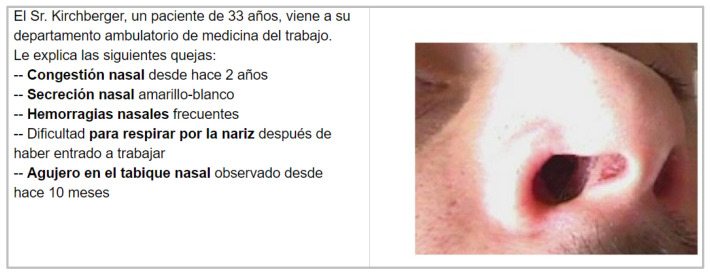
Examples of real cases in the CASUS/virtual patient tool. The tool provides real patient images to provide students with useful visual data to put them in context. Taken from CASUS online. Retrieved from: https://networm.casus.net/pmw2/app/cardtop2.html?docid=1535088447193, https://networm.casus.net/pmw2/app/cardtop2.html?docid=1535088446844 (accessed on 11 September 2021). Translation of the first case to English: Yesterday I went to the obstetrician, and he did an ultrasound and tests, do you want to see the results? Question: What is the gestational age of the fetus (e.g., 4 weeks = 2 weeks after conception), based on the ultrasound and lab results. Multiple choice answer A: 4 weeks, B: 5 weeks, C: 6 weeks, D: 7 weeks. Translation of the second case to English: Mr. Kirchberger, a 33-year-old patient, comes to your outpatient occupational medicine department. He explains to you the following complaints: - nasal congestion for two years, - yellow-white nasal discharge, - frequent nosebleeds - difficulty breathing through the nose after coming to work, - hole in the nasal septum observed in the last 10 months.

**Figure 5:**
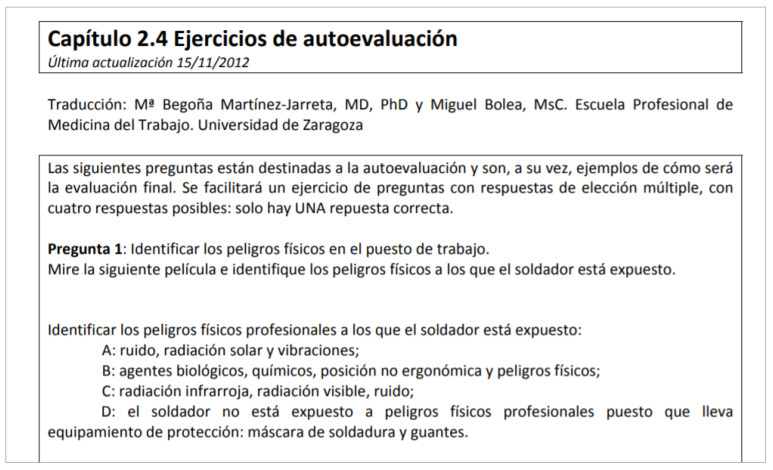
Example of one of the self-assessment documents created for the students who used the EMUTOM module. Each of the subtopics in each topic contains a self-assessment document for students to test their knowledge and check their answers without the help of a tutor. Extracted from “emutom”, by Braeckman, 2012, available at: http://www.emutom.eu/files/chapter2/Capitulo%202.4%C2%A0Ejercicios%C2%A0de%C2%A0autoevaluaci%C3%B3n%C2%A0.PDF (accessed on 11 September 2021). Translation to English. Chapter 2.4. Self-assessment exercises. Last updated 15 November 2012. Translation: Mª Begoña Martínez-Jarreta, MD, PhD and Miguel Bolea, MsC. Professional School of Occupational Medicine. University of Zaragoza. The following questions are intended for self-assessment and are, at the same time, examples of what the final assessment will be like. A multiple-choice question exercise will be provided, with four possible answers: there is only ONE correct answer. Question 1: Identify physical hazards in the workplace. Watch the following film and identify the physical hazards to which the welder is exposed. A: noise, solar radiation and vibrations; B: biological agents, chemicals, non-ergonomic position and physical hazards; C: infrared radiation, visible radiation, noise; D: the welder is not exposed to occupational physical hazards as he/she is wearing protective equipment: welding mask and gloves.

A longitudinal design was used to assess the attitudes of students who completed the course (but before taking the final exam) of Occupational Medicine as part of their medical degree during three consecutive academic years (2015–2016, 2016–2017 and 2017–2018) at the University of Zaragoza (Spain). This optional subject (with five ECTS) was taught with a combination of two different teaching innovative tools, namely, EMUTOM (Figure 4) and virtual patient platform/CASUS (Figure 5), along with traditional lectures. The total sample for the three academic years was 428, which represented a response rate of 99.3% of the total number of students enrolled. This sample was used to validate the questionnaire that was adapted from the questionnaire used by Smits and Verbeek.

Moreover, we assessed the attitudes towards Occupational Medicine as part of a medical degree during the 2017–2018 academic year at the University of Castilla-La Mancha (in Albacete, Spain) before and after completing the course. This subject is not optional but obligatory at this university (with three ECTS) and taught using both the virtual patient platform/CASUS and traditional lectures. The tool EMUTOM was not included as a teaching method. A total of 98 students from the University of Castilla-La Mancha completed the questionnaire of Smits and Verbeek, which had a response rate of 98% of the total students enrolled.

### 2.2. Changes in Attitudes towards Occupational Medicine (before and after Completing the Course)

Additionally, during the 2017–2018 academic year, 111 students from the University of Zaragoza and 98 students from Castilla-La Mancha filled in the questionnaire before and after completing the classes to evaluate possible changes in attitudes.

In both universities, the questionnaire in Spanish was uploaded to the course management system (Moodle section) for the Occupational Medicine subject. An e-mail was sent to the students informing them how to access and fill in the questionnaire and highlighting the need to complete all items. Students could either fill it in from a computer and print it out or print it out without filling it in and complete it by hand. In both cases, the questionnaire was freely and anonymously handed in at the secretary’s office of the university department.

The present study obtained the approval of the regional Ethics Committee of Aragon (CEICA).

## 3. Results

### 3.1. Reliability of the Questionnaire for the Evaluation of Attitude towards Occupational Medicine

To measure the reliability, an analysis of internal consistency and discrimination ability of the items was carried out. The Cronbach’s alpha parameter was used for this purpose, yielding a value of 0.772, which is considered acceptable. However, the first and second items were eliminated because the values in the corrected item-total correlation were lower than 0.2 (−0.012 and 0.199, respectively). Eliminating these items increased the Cronbach’s Alpha from 0.772 to 0.809, which can be considered good. Table S1 shows the data resulting from the reliability study of the items after the above-mentioned elimination (N = 428). The corrected item-total correlation values were greater than 0.2, and the Cronbach’s alpha value did not increase with the removal of any other items. Therefore, the internal consistency is good.

### 3.2. Factor Analysis of the Questionnaire of Attitude towards Occupational Medicine

The factors or dimensions identified in the baseline questionnaire are as follows:

Factor 1 (“interest and importance of training in Occupational Medicine”) represents 17.13% of the total variability. In this factor, the following reasons have high weights: “Return to work after illness forms an important part of rehabilitation” (Actm24); “It is important to diagnose an illness as an occupational disease” (Actm25); “As an occupational physician you need trust between you and your patient” (Actm26); “The kind of work a patient is doing forms an important part of the medical history” (Actm27); “As a physician, you will always know what kind of job your patient holds” (Actm28); “Every physician should report occupational diseases” (Actm210).

Factor 2 (“importance and need for scientific research in Occupational Medicine”) represents 14.71% of the total variability. In this factor, the following reasons have high weights: “The job of the occupational physician is challenging” (Actm1); “I am very much attracted to the preventive aspects of occupational health” (Actm2), “Occupational disability is an interesting topic to study” (Actm3); “From the clinical point of view, occupational diseases are not very interesting (reversed coding)” (Actm21).

Factor 3 (“duties of doctors in their professional practice in detecting occupational diseases and problems of occupational origin”) accounts for 10.89% of the total variability. In this factor, the following reasons have high weights: “There is still a lot to be discovered about occupational diseases” (Actm22); “There is still a lot to be discovered about return to work after illness” (Actm23).

Dimension or Factor 4 (“importance/interest in social, ethical and deontological aspects associated with patients’ work-related problems”) accounts for 10.73% of the total variability. In this factor, the following reasons have high weights: “If you have a certain illness, it is important to take this into consideration already when choosing a career” (Actm29); “With every patient, the doctor looks at the complaints but also at its consequences for functioning in daily life and at work” (Actm211); “It is a problem that, as an occupational physician, one is not independent from the employer” (Actm212).

Table S2 specifies the items that are part of the attitude questionnaire and their factorial saturation in the respective factors, corresponding to and verifying the proposed factorial structure (N = 428). The results of the factorial saturations for items on the attitude towards Occupational Medicine questionnaire endorsed the factorial structure. Factor analysis tries to identify common factors among variables; thus, it is convenient that the variables included in the model were well intercorrelated. In the matrix of correlations revealed previously, we observed that our items were well correlated with each other, as all Pearson’s correlation coefficients were significant.

To choose the number of factors, the size of the eigenvalue is usually used. The factors with the largest eigenvalues were selected, that is, according to Kaiser’s criterion [11], the factors with eigenvalues greater than one. In our case, this applied to the first four. These four factors explained 53% of the variability (results not shown).

In the factorial model, it is essential to choose the number of factors of the model that explain the maximum variability while fulfilling the principle of parsimony. In this case, the four factors were also finally selected based on the following criteria: the percentage of variability explained by the four factors is sufficient, the sedimentation graph, the percentage of variability explained by each variable is reasonable and the interpretation of the factors has theoretical coherence. Taking into account the above-mentioned findings, these four factors were used.

To ensure adequate factors in the factorial analysis, besides high correlations between variables, an adequate KMO index should be achieved. The present sample resulted in a value of 0.839, which is a good value for factor analysis. Bartlett’s test of sphericity was also conducted, and the results were significant with a *p*-value of 0.000.

### 3.3. Attitudes towards Occupational Medicine after Completing the Course

The following data are the results obtained on the attitude towards the subject of Occupational Medicine shown by students in the 2015–2016, 2016–2017 and 2017–2018 academic years. The key data observed are summarized below, and for a better understanding, they are grouped into three categories (Table 1).

Regarding Occupational Medicine as a specialty for their possible future professional practice, 42.5% of the students indicated that they “agree” or “strongly agree” that practicing as an occupational physician is a professional option for them, and 41.8% of students stated that they “agreed” or “strongly agreed” that practicing as an occupational physician was an interesting career option for them.

Concerning the importance of the contents, competences and basic skills in Occupational Medicine and of training in Occupational Medicine for practicing as a doctor, 99.3% of students expressed that they “agree” or “strongly agree” that identifying/diagnosing a disease as an occupational disease is important. A total of 98.8% of the students expressed that they “agree” or “strongly agree” that the type of work that a patient is undertaking or has undertaken should be part of his/her medical record. A total of 98.6% of students indicated that they “agree” or “strongly agree” that, as an occupational physician, it is necessary to establish a doctor–patient relationship based on trust, and 97.9% of the students indicated that they “agree” or “strongly agree” that the doctor should always ask the patient and try to know what kind of work his/her patient is involved in. A total of 96.7% of the students stated that they “agree” or “strongly agree” that returning to work after illness is an important aspect of rehabilitation for patients, and 95.6% of students indicated that they “agree” or “strongly agree” that when a doctor treats a patient, he/she should also consider the consequences that his/her health problems may have on his/her socio-occupational life. A total of 94.1% of students expressed that they “agree” or “strongly agree” that the training and study of occupational diseases were important for them, and 93.7% of the students said that they “agreed” or “strongly agreed” that any practicing doctor should be involved in the reporting of occupational diseases. A total of 93.2% of the students stated that they “agree” or “strongly agree” that occupational diseases are an important type of pathology from a medical perspective. A total of 90.4% of students “agreed” or “strongly agreed” that studying occupational disabilities was interesting to them, and 84.3% of the students indicated that they “agreed” or “strongly agreed” that the fact that an individual suffers from a certain illness should be considered when they choose an occupation. A total of 79.4% of the students indicated that they “agree” or “strongly agree” that it is important for the occupational physician to be able to advise companies on occupational health. A total of 79% of students indicated that they “agree” or “strongly agree” that returning to work after sick leave is an issue on which there is still a lot of research and work to be completed, and 60.7% of students said that they “agree” or “strongly agree” that they are attracted to the preventive aspects of occupational health.

Finally, in terms of industrial relations and recruitment of the occupational physician, 80.3% of the students indicated that they “agree” or “strongly agree” that the occupational physician’s contractual dependence on the employer can create a problem.

### 3.4. Changes in Attitudes towards Occupational Medicine (before and after Completing the Course)

Table 2 shows the results obtained on the attitudes towards Occupational Medicine by students in the 2017–2018 academic year before and after completing the subject, respectively.

Before classes of Occupational Medicine had started, 34.5% strongly agreed with the statement “I find it interesting to study occupational diseases”. After completing the subject, this percentage rose to 56.6%. Similarly, “Advising companies about healthy work is attractive to me” was reported by 60.3% of the respondents at the beginning vs. 75.7% after finishing the subject.

A total of 13.7% strongly agreed with the statement “From the clinical point of view, occupational diseases are not very interesting”. This percentage decreased by up to 4.5% after finishing the course.

A total of 60.4% of the respondents strongly agreed with the statement “There is still a lot to be discovered about occupational diseases”. After finishing the subject, this percentage was 82.9%.

The statement “As an occupational physician you need trust between you and your patient” obtained a percentage of 63.8% at the beginning of the course and 79.6% after finishing the subject.

Likewise, 69% of the participants responded that they strongly agreed with the statement “The kind of work a patient is doing forms an important part of the medical history” prior to the course, and this percentage increased to 81.6% after completing it.

Finally, for the statement “With every patient, the doctor looks at the complaints but also at its consequences for functioning in daily life and at work”, the percentage of respondents who stated “strongly agree” increased from 56.9% at the beginning to 73.7% after completing the subject.

Although similar results were found in the sample analyzed in the University of Castilla-La Mancha, a generally more positive attitude towards Occupational Medicine was found in the University of Zaragoza before and after taking the course (Tables S3–S6).

### 3.5. Open Questions

Generally, students reported the absence of practical learning throughout their medical degree and the need to learn with more practical content.

In relation to blended learning, students indicated that online cases (CASUS) and e-learning (EMUTOM) should be more common, and students considered the blended learning format more attractive since the use of PowerPoint was the only teaching method in other subjects.

Occupational Medicine was seen as an unattractive subject in many cases, but students stated that the blended learning implemented in this course made it much more attractive, helping them to change their attitude after taking it.

## 4. Discussion

The present study aimed to determine whether the teaching and training of Occupational Medicine among medical students from two Spanish universities could positively change their attitudes towards this specialty. We found a significant change in students’ attitudes after they had taken the Occupational Medicine course in both universities, with students perceiving the contents of the subject as much more important and necessary than before starting the course. Both their interest in the subject and their perception of the importance of the ethical issues involved increased.

Similar to other studies in upper-income countries, our results show that Occupational Medicine was not a priori an attractive subject, and students maintained their position of neutrality when deciding whether it was a specialty that they, individually, would like to embrace in the future [12,13,14]. In our study, a low percentage of students in the 5th year of medicine considered Occupational Medicine as a potential medical specialty that they may choose. Moreover, they did not look favorably on the contractual dependence of the occupational physician on the employer being provided with the physician’s services. These results can be explained by the fact that Occupational Medicine has high administrative content and is far removed from the classic practice of the medical and medical-surgical specialties that are most appreciated as a classic professional outlet (cardiology, surgery, internal medicine, etc.).

Despite the above, the importance that the students of this study attributed to training and the acquisition of competences and skills in Occupational Medicine was very high, and they perceived this training as fundamental for their future professional performance as doctors in any specialty.

Some of the items on which more than 90% of the students in the sample said they “agreed” or “strongly agreed” are objectives traditionally identified in Spain and Europe as “pending” and difficult to achieve [8,12]. These results are related to the items: “It is important to diagnose an illness as an occupational disease”, “The kind of work a patient is doing forms an important part of the medical history” and “Every physician should report occupational diseases”.

Additionally, as suggested by the responses to the open questions, the positive attitudes of the students collected after completing the course of Occupational Medicine could be due to the blended learning format. The use of innovative teaching tools used in teaching might have facilitated an interest in the subject of Occupational Medicine and the change in attitude before and after taking the course.

Only a few studies have assessed changes in the attitudes of medical students towards Occupational Medicine/Occupational Health, a subject that some European medical schools do not include at all, or if they do, only several hours are devoted to this subject [8,12]. In line with our results, some previous studies have shown an improvement in students’ attitudes towards and competencies in Occupational Medicine when various learning methods, including case studies, small-group learning, interactive large-group teaching, field activities and e-learning, were applied [15,16,17] or when blended learning was applied but in the subject of Family Medicine [13]. Conversely, in the field of Occupational Medicine but among Physiotherapy students, the study of Eckler et al. did not offer a general recommendation favoring either the classical setting or blended learning for improving cognitive learning outcomes, although students’ self-reports on the affective domain indicated a preference for blended learning [18]. Similarly, in the study of Smits et al., case-based e-learning did not improve the attitudes of medical students towards occupational health when compared with non-case-based textbook learning [14]. The results of this study cannot be compared with ours because of differences in the sample characteristics. For example, medical students were from different academic years (second-year vs. fifth-year medical students). Students in the first two years are eager for clinical training, which is in line with their expectations of a doctor’s role. Hence, they might not be prepared to understand the specialties of Social Medicine (Occupational Medicine, Preventive Medicine and Public Health, and Forensic Medicine). Moreover, students are not equally motivated if the subject is optional or compulsory. There were also differences in the number of credits dedicated to Occupational Medicine, as well as differences in the syllabus and teaching methods. Variables such as the professor’s teaching experience, communication skills and motivational capacity are difficult to measure but can also influence a possible change in attitude.

It could be argued that the positive attitudes observed after taking this course could be explained by the fact that students did not really know what Occupational Medicine was and were ignorant about all of the competences provided by this medical field. Nonetheless, the significant improvement in the students’ attitudes towards Occupational Medicine was mainly observed in the University of Zaragoza compared to the University of Castilla-La Mancha. The detected higher positive attitudes in students from the University of Zaragoza could be explained by several factors. Firstly, Occupational Medicine is an optional subject in Zaragoza, whereas it is an obligatory subject in Castilla-La Mancha; thus, as previously explained, students had freely chosen this subject in Zaragoza and therefore could be more prone to already like the subject. Secondly, in the University of Zaragoza, both virtual patients/CASUS [19] and EMUTOM were applied rather than the use of virtual patients only, as is the case in Castilla-La Mancha. Differences between the two Universities could also be explained by other factors, such as personal factors (e.g., professors who were responsible for classes in the University of Zaragoza could have been more enthusiastic than those in Castilla-La Mancha).

In the present investigation, the students could work with both virtual patients and the EMUTOM tool whenever they preferred to be at home and from any computer with their unique passwords.

On the contrary, in the study of Smits et al., students were grouped in a classroom with computers to work with cases during class hours. This means that they detract several of the main benefits of these innovative resources, such as autonomy—freedom of time and place, etc. [14].

Our results suggest that after completing the course, students took Occupational Medicine into great consideration, perceiving the detection of occupational diseases as a medical obligation, as well as placing a high value on the social aspects of problems of occupational origin. These results are corroborated by the open answers provided in the questionnaire, which shows that the interest generated by the subject and the recognition of its importance were influenced by the modality in which it was taught (blended learning format) and the use of innovative tools.

To our knowledge, this is the first study assessing the attitudes and changes in attitudes towards Occupational Medicine in Spain and one of the first worldwide. Using either one or both systems—online virtual patient platforms (CASUS) and/or EMUTOM—as a blended learning model at two Spanish Medical Universities, we studied the attitudes towards Occupational Medicine in 526 students. We achieved an excellent response rate (over 98%), which avoids selection bias. Moreover, we explored three academic years (2015–2016, 2016–2017 and 2017–2018) before the COVID-19 pandemic, during which e-learning methods were an obligation rather than an optional tool.

Nevertheless, there are some limitations that should be acknowledged. Firstly, not all data were available during the three academic years. In fact, information before and after completing the course was available for just one academic year. Additionally, we did not have two groups that we could compare to have a better understanding of the results (i.e., one group takes the course using online educational materials with opportunities for online interaction with traditional place-based classroom methods, and another one learns using a more traditional method). Moreover, we have to account for possible social desirability in this study. Although questionnaires were filled in anonymously, medical students may have selected socially acceptable options rather than the “true” answers. Finally, we included some open questions that allowed us to obtain some extra information about the perceptions of the blended learning method, including the EMUTOM and the CASUS tool, as well as the students’ perceptions of Occupational Medicine. However, these open questions were not validated. In addition, we did not have a control group (students taught with traditional methods vs. those taught using a blended learning methodology) that allowed us to compare the two methods and to draw conclusions about which strategy achieved better results.

It is necessary to increase the scientific evidence and to carry out new studies in this regard to fully characterize the factors that could be behind this desirable change in attitude towards Occupational Medicine (i.e., the blended learning method, the learning process of the competences of the subject, the enthusiasm of the lecturer or a combination of all of them). Since the results cannot be generalized, future research should be carried out to compare different teaching methods (i.e., traditional vs. blended learning vs. hybrid vs. flipped courses) and assess the attitudes of students before and after taking the course, preferably in students in the last years of Medicine, who could be more motivated to learn the specialties of Social Medicine.

## 5. Conclusions

On average, Occupational Medicine is not an attractive subject or profession to medical students in Spain. However, this study showed that teaching and training medical students on Occupational Medicine can change this negative perception. Despite these previous negative or neutral attitudes towards Occupational Medicine, significant differences were found in their attitude in 10 of the 18 items before and after completing the Occupational Medicine course. This significant change will allow students to recognize the importance of training in Occupational Medicine and the acquisition of specific competences and skills to adequately practice medicine, regardless of which medical specialty is finally chosen. Further research is needed for in-depth evaluation to identify the main determinants (i.e., the blended learning method, the learning process of the competences of the subject, the enthusiasm of the lecturer or a combination of all of them) that induce a significant change in medical students’ attitudes towards Occupational Medicine.

## Figures and Tables

**Table 1:** Results of the attitude questionnaire after studying Occupational Medicine at the University of Zaragoza during the 2015–2016, 2016–2017 and 2017–2018 academic years (N = 428).

Items	%						Median	Mean	SD
	Strongly Disagree	Disagree	Neutral	Agree	Strongly Agree	Ns/Nc			
A career in Occupational Medicine (six statements)									
1. The job of the occupational physician is challenging (Actm1)	7.9	19.6	29.9	34.6	7.9	0.0	3.0	3.1	1.08
2. I am very much attracted to the preventive aspects of occupational health (Actm2)	2.1	16.8	20.3	51.4	9.3	0.0	4.0	3.5	0.95
3. Occupational disability is an interesting topic to study (Actm3)	0.0	2.8	6.8	69.6	20.8	0.0	4.0	4.1	0.62
4. One of my options is to become an occupational physician (Actm4)	8.2	18.9	30.8	34.1	7.7	0.2	3.0	3.1	1.07
5. I find it interesting to study occupational diseases (Actm5)	0.2	1.4	3.7	48.8	45.3	0.5	4.0	4.4	0.65
6. Advising companies about healthy work is attractive to me (Actm6)	1.4	9.8	9.3	28.5	50.9	0.0	5.0	4.2	1.05
Occupational Medicine as an interesting medical specialty (11 statements)									
1. From the clinical point of view, occupational diseases are not very interesting (reversed coding) (Actm21)	0.5	1.6	4.7	40.9	52.3	0.0	5.0	4.4	0.70
2. There is still a lot to be discovered about occupational diseases (Actm22)	0.5	2.1	11.4	44.9	41.1	0.0	4.0	4.2	0.77
3. There is still a lot to be discovered about return to work after illness (Actm23)	0.0	2.3	18.7	54.7	24.3	0.0	4.0	4.0	0.72
4. Return to work after illness forms an important part of rehabilitation (Actm24)	0.0	0.5	2.8	37.4	59.3	0.0	5.0	4.6	0.58
5. It is important to diagnose an illness as an occupational disease (Actm25)	0.5	0.0	0.2	25.7	73.6	0.0	5.0	4.7	0.51
6. As an occupational physician you need trust between you and your patient (Actm26)	0.5	0.0	0.7	29.0	69.6	0.2	5.0	4.7	0.54
7. The kind of work a patient is doing forms an important part of the medical history (Actm27)	0.0	0.5	0.7	18.7	80.1	0.0	5.0	4.8	0.46
8. As a physician, you will always know what kind of job your patient holds (Actm28)	0.0	0.5	0.7	30.4	67.5	0.9	5.0	4.7	0.52
9. If you have a certain illness, it is important to take this into consideration already when choosing a career (Actm29)	0.5	4.2	10.0	50.7	33.6	0.9	4.0	4.1	0.80
10. Every physician should report occupational diseases (Actm210)	0.0	1.9	3.5	36.2	57.5	0.9	5.0	4.5	0.66
11. With every patient, the doctor looks at the complaints but also at its consequences for functioning in daily life and at work (Actm211)	0.0	0.9	2.3	29.7	65.9	1.2	5.0	4.6	0.58
Role and position of the occupational physician (one statement)									
12. It is a problem that, as an occupational physician, one is not independent from the employer (Actm212)	0.2	4.7	13.1	45.3	35.0	1.6	4.0	4.1	0.83

**Table 2:** Results of the attitude questionnaire before (B) and after (A) studying Occupational Medicine in the 2017–2018 academic year (N = 111).

	Percentage (%)																	
	Strongly Disagree		Disagree		Neutral		Agree		Strongly Agree		Ns/Nc		Median		Mean		SD	
	B	A	B	A	B	A	B	A	B	A	B	A	B	A	B	A	B	A
1. The job of the occupational physician is challenging (Actm1)	17.2	11.2	20.7	27.0	43.1	34.9	19.0	24.3	0.0	2.6	0.0	0.0	3.0	3.0	2.6	2.8	1.0	1.02
2. I am very much attracted to the preventive aspects of occupational health (Actm2)	1.7	2.6	20.7	14.5	24.1	19.1	48.3	52.0	5.2	11.8	0.0	0.0	4.0	4.0	3.3	3.6	0.9	0.97
3. Occupational disability is an interesting topic to study (Actm3)	0.0	0.0	3.4	2.6	27.6	8.6	53.4	65.8	10.3	23.0	5.2	0.0	4.0	4.0	3.7	4.1	0.7	0.64
4. One of my options is to become an occupational physician (Actm4)	5.2	5.3	15.5	13.2	27.6	30.9	48.3	40.8	3.4	9.9	0.0	0.0	4.0	4.0	3.3	3.4	1.0	1.01
5. I find it interesting to study occupational diseases (Actm5)	0.0	0.0	0.0	1.3	0.0	0.7	65.5	40.1	34.5	56.6	0.0	1.3	4.0	5.0	4.3	4.5	0.5	0.59
6. Advising companies about healthy work is attractive to me (Actm6)	1.7	1.3	1.7	0.7	0.0	0.0	36.2	22.4	60.3	75.7	0.0	0.0	5.0	5.0	4.5	4.7	0.8	0.64
Occupational Medicine as an interesting medical specialty (11 statements)																		
1. From the clinical point of view, occupational diseases are not very interesting (reversed coding) (Actm21)	0.0	1.3	3.4	0.7	10.3	2.6	39.7	28.9	46.6	66.4	0.0	0.0	4.0	5.0	4.3	4.6	0.8	0.70
2. There is still a lot to be discovered about occupational diseases (Actm22)	0.0	1.3	1.7	3.9	37.9	11.8	46.6	44.7	13.8	38.2	0.0	0.0	4.0	4.0	3.7	4.1	0.7	0.87
3. There is still a lot to be discovered about return to work after illness (Actm23)	0.0	0.0	8.6	3.9	22.4	17.8	53.4	50.0	15.5	28.3	0.0	0.0	4.0	4.0	3.8	4.0	0.8	0.79
4. Return to work after illness forms an important part of rehabilitation (Actm24)	0.0	0.0	0.0	1.3	3.4	2.6	55.2	27.0	41.4	69.1	0.0	0.0	4.0	5.0	4.4	4.6	0.6	0.60
5. It is important to diagnose an illness as an occupational disease (Actm25)	0.0	1.3	0.0	0.0	0.0	0.0	31.0	21.1	69.0	77.6	0.0	0.0	5.0	5.0	4.7	4.7	0.5	0.60
6. As an occupational physician you need trust between you and your patient (Actm26)	0.0	1.3	1.7	0.0	0.0	0.0	34.5	19.1	63.8	79.6	0.0	0.0	5.0	5.0	4.6	4.8	0.6	0.59
7. The kind of work a patient is doing forms an important part of the medical history (Actm27)	0.0	0.0	0.0	1.3	5.2	0.0	25.9	17.1	69.0	81.6	0.0	0.0	5.0	5.0	4.6	4.8	0.6	0.50
8. As a physician, you will always know what kind of job your patient holds (Actm28)	0.0	0.0	0.0	0.7	1.7	0.0	41.4	30.9	56.9	65.8	0.0	2.6	5.0	5.0	4.6	4.7	0.5	0.52
9. If you have a certain illness, it is important to take this into consideration already when choosing a career (Actm29)	1.7	0.0	3.4	1.3	6.9	5.9	53.4	52.0	34.5	38.2	0.0	2.6	4.0	4.0	4.2	4.3	0.8	0.65
10. Every physician should report occupational diseases (Actm210)	0.0	0.0	0.0	3.3	6.9	2.0	46.6	32.9	46.6	59.2	0.0	2.6	4.0	5.0	4.4	4.5	0.6	0.70
11. With every patient, the doctor looks at the complaints but also at its consequences for functioning in daily life and at work (Actm211)	0.0	0.0	0.0	0.7	1.7	0.0	41.4	23.0	56.9	73.7	0.0	2.6	5.0	5.0	4.6	4.7	0.5	0.48
Role and position of the occupational physician (one statement)																		
12. It is a problem that, as an occupational physician, one is not independent from the employer (Actm212)	1.7	0.0	1.7	5.3	15.5	13.8	48.3	42.1	32.8	36.2	0.0	2.6	4.0	4.0	4.1	4.1	0.8	0.85

## Data Availability

The raw data supporting the conclusions of this article will be made available by the authors upon request, without undue reservation.

## Supplementary Materials

The following are available online at https://www.mdpi.com/article/10.3390/ijerph19020878/s1, Table S1: Reliability analysis of the items of the subject attitude questionnaire with the first two items removed (N = 428). Table S2: Items and factorial saturations of the Occupational Medicine subject attitude questionnaire (N = 428). Table S3: Results of the attitude questionnaire before and after studying Occupational Medicine at the University of Castilla la Mancha in the 2017–2018 academic year (N = 98). Table S4: Results of the attitude questionnaire before and after studying Occupational Medicine at the University of Castilla la Mancha in the 2017–2018 academic year (N = 98). Table S5: Differences in attitudes towards Occupational Medicine between Zaragoza and Castilla la Mancha before taking the course. Table S6: Differences in attitudes towards Occupational Medicine between Zaragoza and Castilla la Mancha after taking the course.
