# Supplementary material for: Changing Attitudes towards Occupational Medicine with Blended Learning Methods Is Possible among Medical Students in Spain: A Longitudinal Study
Source: Int J Environ Res Public Health. 2022 Jan 13;19(2):878. doi: 10.3390/ijerph19020878 (PMC8775897; doi:10.3390/ijerph19020878)
Supplement: Supplementary file 1 [file ijerph-19-00878-s001.zip › ijerph-1498831-supplementary.pdf]

SUPPLEMENTARY MATERIAL

Table S1: Reliability analysis of the items of the subject attitude questionnaire with the first two items removed (N=428)

	Mean of the scale if the element is removed	Variance of the scale if the element is removed	Corrected item-total correlation	Squared multiple correlation	Cronbach's alpha if the element is removed
**Actm3**	65,26	30,658	0,308	0,178	0,805
**Actm4**	66,20	27,381	0,411	0,356	0,803
**Actm5**	64,95	29,303	0,501	0,391	0,793
**Actm6**	65,16	26,358	0,540	0,426	0,789
**Actm21**	64,92	28,451	0,568	0,389	0,788
**Actm22**	65,11	29,389	0,374	0,339	0,801
**Actm23**	65,34	29,531	0,391	0,348	0,800
**Actm24**	64,79	29,863	0,465	0,301	0,796
**Actm25**	64,63	30,138	0,489	0,414	0,796
**Actm26**	64,67	30,026	0,483	0,359	0,796
**Actm27**	64,56	30,502	0,495	0,405	0,797
**Actm28**	64,68	31,107	0,306	0,194	0,805
**Actm29**	65,20	29,363	0,368	0,232	0,802
**Actm210**	64,84	29,973	0,375	0,207	0,801
**Actm211**	64,73	30,026	0,432	0,261	0,798
**Actm212**	65,22	29,794	0,294	0,161	0,808

1: the first two items have been removed and all corrected item-total correlation values are above the 0.2 cut-off point.

Table S2: Items and factorial saturations of the Occupational Medicine subject attitude questionnaire. Highlighted values are those that saturate the factor. rotation method: VARIMAX (N=428).

	FACTOR WEIGHTS			
	**Factor 1**	**Factor 2**	**Factor 3**	**Factor 4**
**Actm3**	0,114	**0,585**	0,189	-0,198
**Actm4**	-0,108	**0,708**	0,014	0,404

**Actm5**	0,278	**0,755**	-0,001	0,007
**Actm6**	0,181	**0,635**	-0,020	0,436
**Actm21**	0,354	**0,578**	0,267	0,064
**Actm22**	0,208	0,107	**0,772**	0,002
**Actm23**	0,083	0,080	**0,836**	0,172
**Actm24**	**0,458**	0,149	0,328	0,206
**Actm25**	**0,728**	0,182	0,218	-0,053
**Actm26**	**0,662**	0,223	0,073	0,091
**Actm27**	**0,747**	0,109	0,150	0,070
**Actm28**	**0,572**	-0,053	-0,115	0,288
**Actm29**	0,074	0,147	0,051	**0,745**
**Actm210**	**0,410**	0,158	0,246	0,103
**Actm211**	0,420	0,115	-0,024	**0,507**
**Actm212**	0,133	-0,064	0,283	**0,572**

Table S3. Results of the Attitude questionnaire **before (B) and after (A)** studying Occupational Medicine in the University of Castilla la Mancha in the academic year 2017-2018 (N=98)

	**Percentage (%)**																	
	**Strongly disagree (1)**		**Disagree (2)**		**Neutral (3)**		**Agree (4)**		**Strongly agree (5)**		**Ns/Nc**		**Median**		**Mean**		**SD**	
	**B**	**A**	**B**	**A**	**B**	**A**	**B**	**A**	**B**	**A**	**B**	**A**	**B**	**A**	**B**	**A**	**B**	**A**
**1. The job of the occupational physician is challenging (Actm1)**	35,7	20,0	35,7	37,8	23,5	28,9	3,1	8,9	2,0	4,4	0,0	0,0	2,0	2,0	2,0	2,4	0,95	1,05
**2. I am very much attracted to the preventive aspects of occupational health (Actm2)**	15,3	8,9	22,4	11,1	31,6	28,9	27,6	44,4	2,0	6,7	1,0	0,0	3,0	4,0	2,8	3,3	1,08	1,05
**3. Occupational disability is an interesting topic to study (Actm3)**	9,2	5,6	15,3	5,6	37,8	7,8	34,7	60,0	2,0	20,0	1,0	1,1	3,0	4,0	3,1	3,8	0,98	1,00
**4. One of my options is to become an occupational physician (Actm4)**	11,2	10,0	33,7	17,8	35,7	26,7	16,3	36,7	1,0	8,9	2,0	0,0	3,0	3,0	2,6	3,2	0,93	1,13

**5. I find it interesting to study occupational diseases (Actm5)**	1,0	0,0	4,1	3,3	15,3	2,2	63,3	61,1	15,3	33,3	1,0	0,0	4,0	4,0	3,9	4,2	0,75	0,66
**6. Advising companies about healthy work is attractive to me (Actm6)**	0,0	0,0	0,0	1,1	6,1	1,1	73,5	43,3	20,4	54,4	0,0	0,0	4,0	5,0	4,1	4,5	0,50	0,59
**Occupational medicine as an interesting medical specialty (11 statements)**																		
**1. From the clinical point of view, occupational diseases are not very interesting (reversed coding) (Actm21)**	0,0	0,0	2,0	2,2	16,3	5,6	62,2	52,2	19,4	40,0	0,0	0,0	4,0	4,0	4,0	4,3	0,67	0,68
**2. There is still a lot to be discovered about occupational diseases (Actm22)**	0,0	0,0	5,1	3,3	34,7	10,0	46,9	54,4	13,3	32,2	0,0	0,0	4,0	4,0	3,7	4,2	0,77	0,73
**3. There is still a lot to be discovered about return to work after illness (Actm23)**	0,0	0,0	5,1	4,4	29,6	20,0	51,0	60,0	13,3	15,6	1,0	0,0	4,0	4,0	3,7	3,9	0,76	0,72
**4. Return to work after illness forms an important part of rehabilitation (Actm24)**	0,0	1,1	0,0	0,0	3,1	3,3	66,3	58,9	29,6	36,7	1,0	0,0	4,0	4,0	4,3	4,3	0,51	0,64
**5. It is important to diagnose an illness as an occupational disease (Actm25)**	0,0	0,0	0,0	0,0	6,1	3,3	62,2	51,1	31,6	45,6	0,0	0,0	4,0	4,0	4,3	4,4	0,56	0,56
**6. As an occupational physician you need trust between you and your patient (Actm26)**	2,0	0,0	1,0	0,0	6,1	3,3	50,0	32,2	40,8	64,4	0,0	0,0	4,0	5,0	4,3	4,6	0,79	0,55
**7. The kind of work a patient is doing forms an important part of the medical history (Actm27)**	1,0	0,0	0,0	0,0	3,1	2,2	41,8	33,3	54,1	64,4	0,0	0,0	5,0	5,0	4,5	4,6	0,66	0,53
**8. As a physician, you will always know what kind of job your patient holds (Actm28)**	0,0	0,0	1,0	0,0	6,1	4,4	52,0	47,8	37,8	47,8	3,1	0,0	4,0	4,0	4,3	4,4	0,64	0,58
**9. If you have a certain illness, it is important to take this into consideration already when choosing a career (Actm29)**	0,0	0,0	2,0	1,1	9,2	6,7	58,2	63,3	27,6	28,9	3,1	0,0	4,0	4,0	4,1	4,2	0,67	0,60
**10. Every physician should report**	1,0	0,0	1,0	0,0	13,3	7,8	61,2	46,7	20,4	45,6	3,1	0,0	4,0	4,0	4,0	4,4	0,70	0,63

**occupational diseases (Actm210)**																		
**11. With every patient, the doctor looks at the complaints but also at its consequences for functioning in daily life and at work (Actm211)**	0,0	0,0	1,0	4,4	3,1	4,4	52,0	38,9	40,8	56,7	3,1	0,0	4,0	5,0	4,4	4,5	0,60	0,58
**Role and position of the occupational physician (one statement)**																		
**12. It is a problem that, as an occupational physician, one is not independent from the employer (Actm212)**	1,0	2,2	1,0	5,3	19,4	12,2	50,0	46,7	25,5	36,2	3,1	0,0	4,0	4,0	4,0	4,1	0,78	0,92

Table S4. Results of the Attitude questionnaire **before and after** studying Occupational Medicine in the University of Castilla la Mancha in the academic year 2017-2018 (N=98)

	**Before**			**After**			
**Items**	**Median**	**Mean**	**SD**	**Median**	**Mean**	***SD***	***U Mann-Whitney***
	**A career in occupational medicine (six statements)**						
**1. The job of the occupational physician is challenging (Actm1)**	2,0	2,00	0,95	2,0	2,40	1,05	**0,007**
**2. I am very much attracted to the preventive aspects of occupational health (Actm2)**	3,0	2,78	1,08	4,0	3,29	1,05	**0,001**
**3. Occupational disability is an interesting topic to study (Actm3)**	3,0	3,05	0,98	4,0	3,84	1,00	**0,000**
**4. One of my options is to become an occupational physician (Actm4)**	3,0	2,61	0,93	3,0	3,17	1,13	**0,000**
**5. I find it interesting to study occupational diseases (Actm5)**	4,0	3,89	0,75	4,0	4,24	0,66	**0,000**
**6. Advising companies about healthy work is attractive to me (Actm6)**	4,0	4,14	0,50	5,0	4,51	0,59	**0,000**
	**Occupational medicine as an interesting medical specialty (11 statements)**						
**1. From the clinical point of view, occupational diseases are not very interesting (reversed coding) (Actm21)**	4,0	3,99	0,67	4,0	4,30	0,68	**0,001**
**2. There is still a lot to be discovered about occupational diseases (Actm22)**	4,0	3,68	0,77	4,0	4,16	0,73	**0,000**
**3. There is still a lot to be discovered about return to work after illness (Actm23)**	4,0	3,73	0,76	4,0	3,87	0,72	0,177

**4. Return to work after illness forms an important part of rehabilitation (Actm24)**	4,0	4,27	0,51	4,0	4,30	0,64	0,435
**5. It is important to diagnose an illness as an occupational disease (Actm25)**	4,0	4,26	0,56	4,0	4,42	0,56	**0,042**
**6. As an occupational physician you need trust between you and your patient (Actm26)**	4,0	4,27	0,79	5,0	4,61	0,55	**0,001**
**7. The kind of work a patient is doing forms an important part of the medical history (Actm27)**	5,0	4,48	0,66	5,0	4,62	0,53	0,137
**8. As a physician, you will always know what kind of job your patient holds (Actm28)**	4,0	4,31	0,64	4,0	4,43	0,58	0,185
**9. If you have a certain illness, it is important to take this into consideration already when choosing a career (Actm29)**	4,0	4,15	0,67	4,0	4,20	0,60	0,666
**10. Every physician should report occupational diseases (Actm210)**	4,0	4,02	0,70	4,0	4,38	0,63	**0,000**
**11. With every patient, the doctor looks at the complaints but also at its consequences for functioning in daily life and at work (Actm211)**	4,0	4,37	0,60	5,0	4,52	0,58	0,065
**Role and position of the occupational physician (one statement)**							
**12. It is a problem that, as an occupational physician, one is not independent from the employer (Actm212)**	4,0	4,01	0,78	4,0	4,07	0,92	0,330

Table S5. Differences in attitudes towards Occupational Medicine between Zaragoza and Castilla la Mancha **before** taking the subject

	**Attitudes**						
Items	**Zaragoza (N=111)**			**Castilla la Mancha (N=98)**			**U Mann-Whitney (p-value)**
	Median	***Mean***	***SD***	***Median***	***Mean***	***SD***	
**Actm 1**	3,0	2,64	0,99	2,0	2,00	0,95	**0,000**
**Actm 2**	4,0	3,34	0,93	3,0	2,78	1,08	**0,002**
**Actm 3**	4,0	3,75	0,70	3,0	3,05	0,98	**0,000**
**Actm4**	4,0	3,29	0,96	3,0	2,61	0,93	**0,000**
**Actm5**	4,0	4,34	0,48	4,0	3,89	0,75	**0,000**
**Actm6**	5,0	4,52	0,75	4,0	4,14	0,50	**0,000**
**Actm21**	4,0	4,29	0,79	4,0	3,99	0,67	**0,003**
**Actm22**	4,0	3,72	0,72	4,0	3,68	0,77	0,841
**Actm23**	4,0	3,76	0,82	4,0	3,73	0,76	0,696
**Actm24**	4,0	4,38	0,56	4,0	4,27	0,51	0,181
**Actm25**	5,0	4,69	0,47	4,0	4,26	0,56	**0,000**
**Actm26**	5,0	4,60	0,59	4,0	4,27	0,79	**0,003**

**Actm27**	5,0	4,64	0,58	5,0	4,48	0,66	0,094
**Actm28**	5,0	4,55	0,54	4,0	4,31	0,64	**0,019**
**Actm29**	4,0	4,16	0,83	4,0	4,15	0,67	0,587
**Actm210**	4,0	4,40	0,62	4,0	4,02	0,70	**0,001**
**Actm211**	5,0	4,55	0,54	4,0	4,37	0,60	0,066
**Actm212**	4,0	4,09	0,84	4,0	4,01	0,78	0,432

Table S6. Differences in attitudes towards Occupational Medicine between Zaragoza and Castilla la Mancha **after** taking the subject

	**Attitudes**						
Items	**Zaragoza (N=111)**			**Castilla la Mancha (N=98)**			**U Mann-Whitney (p-value)**
	Median	***Mean***	***SD***	***Median***	***Mean***	***SD***	
**Actm 1**	3,0	2,80	1,02	2,0	2,40	1,05	**0,002**
**Actm 2**	4,0	3,56	0,97	4,0	3,29	1,05	**0,045**
**Actm 3**	4,0	4,09	0,64	4,0	3,84	1,00	0,150
**Actm4**	4,0	3,37	1,01	3,0	3,17	1,13	0,212
**Actm5**	5,0	4,54	0,59	4,0	4,24	0,66	**0,000**
**Actm6**	5,0	4,70	0,64	5,0	4,51	0,59	**0,001**
**Actm21**	5,0	4,59	0,70	4,0	4,30	0,68	**0,000**
**Actm22**	4,0	4,14	0,87	4,0	4,16	0,73	0,732
**Actm23**	4,0	4,03	0,79	4,0	3,87	0,72	0,088
**Actm24**	5,0	4,64	0,60	4,0	4,30	0,64	**0,000**
**Actm25**	5,0	4,74	0,60	4,0	4,42	0,56	**0,000**
**Actm26**	5,0	4,76	0,59	5,0	4,61	0,55	**0,009**
**Actm27**	5,0	4,79	0,50	5,0	4,62	0,53	**0,003**
**Actm28**	5,0	4,66	0,52	4,0	4,43	0,58	**0,002**
**Actm29**	4,0	4,30	0,65	4,0	4,20	0,60	0,152
**Actm210**	5,0	4,52	0,70	4,0	4,38	0,63	**0,027**
**Actm211**	5,0	4,74	0,48	5,0	4,52	0,58	**0,002**
**Actm212**	4,0	4,12	0,85	4,0	4,07	0,92	0,765
